# Current strategies for reproductive management of gilts and sows in North America

**DOI:** 10.1186/2049-1891-6-3

**Published:** 2015-01-31

**Authors:** Robert R Kraeling, Stephen K Webel

**Affiliations:** L&R Research Associates, Watkinsville, GA USA; JBS United Animal Health, Sheridan, IN USA

**Keywords:** Gilts, Management, Nutrition, Reproductive technology, Sows

## Abstract

Many advances in genetic selection, nutrition, housing and disease control have been incorporated into modern pork production since the 1950s resulting in highly prolific females and practices and technologies, which significantly increased efficiency of reproduction in the breeding herd. The objective of this manuscript is to review the literature and current industry practices employed for reproductive management. In particular the authors focus on assisted reproduction technologies and their application for enhanced productivity. Modern maternal line genotypes have lower appetites and exceptional lean growth potential compared to females of 20 yr ago. Thus, nutrient requirements and management techniques and technologies, which affect gilt development and sow longevity, require continuous updating. Failure to detect estrus accurately has the greatest impact on farrowing rate and litter size. Yet, even accurate estrus detection will not compensate for the variability in the interval between onset of estrus and actual time of ovulation. However, administration of GnRH analogs in weaned sows and in gilts after withdrawal of altrenogest do overcome this variability and thereby synchronize ovulation, which makes fixed-time AI practical. Seasonal infertility, mediated by temperature and photoperiod, is a persistent problem. Training workers in the art of stockmanship is of increasing importance as consumers become more interested in humane animal care. Altrenogest, is used to synchronize the estrous cycle of gilts, to prolong gestation for 2–3 d to synchronize farrowing and to postpone post-weaning estrus. P.G. 600® is used for induction of estrus in pre-pubertal gilts and as a treatment to overcome seasonal anestrous. Sperm cell numbers/dose of semen is significantly less for post cervical AI than for cervical AI. Real-time ultrasonography is used to determine pregnancy during wk 3–5. PGF_2α_ effectively induces farrowing when administered within two d of normal gestation length. Ovulation synchronization, single fixed-time AI and induced parturition may lead to farrowing synchronization, which facilitates supervision and reduces stillbirths and piglet mortality. Attendance and assistance at farrowing is important especially to ensure adequate colostrum consumption by piglets immediately after birth. New performance terminologies are presented.

## Introduction

Basic and applied research in physiology, nutrition, genetics, animal behavior, environment and housing over the last 40 yr provided the foundation for development of highly prolific females and various management practices and technologies, which have significantly increased efficiency of reproduction in the breeding herd. An ovulation rate of 20 is not uncommon in contemporary highly prolific females [1]. Therefore, if one assumes a gestation length of 115 d, a lactation length of 21 d, a weaning-to-estrus interval (WEI) of 5 d, 100% conception rate and zero embryonic and preweaning mortality, sows have the potential to farrow 2.6 times/yr and to produce 52 pigs weaned/mated sow/yr. However, due to numerous factors, such as season, nutrition, disease, embryo mortality before d 30 of pregnancy and piglet preweaning mortality, the potential of 52 weaned pigs/mated sow/yr has not been reached. In 2012, the number of liveborn pigs/litter was 11.8–12.3, pigs weaned/mated sow was 10.3–10.5 and the average number of litters/mated female/yr was 2.3 [2, 3]. Thus, the average number of pigs/mated female/yr was approximately 24 in 2012. In 2012, according to PigChamp [2], the average Canadian farrowing rate and total born were 86.6% and 14.0, respectively, and in the U.S.A. they were 83.6% and 13.4, respectively. Comparable data from 2001 for Canada were 74.9% and 11.5 and for the U.S.A. were 69.0% and 11.3. In 2014, Ketchem and Rix [4] reported the following data for the highest 10%, the average and the lowest 30% of producers, respectively: 30.1; 25.3; 21.9 weaned pigs/mated female/yr and 36.3; 32.7; 29.6 total pigs born per litter/mated female/yr.

**Figure 1:**
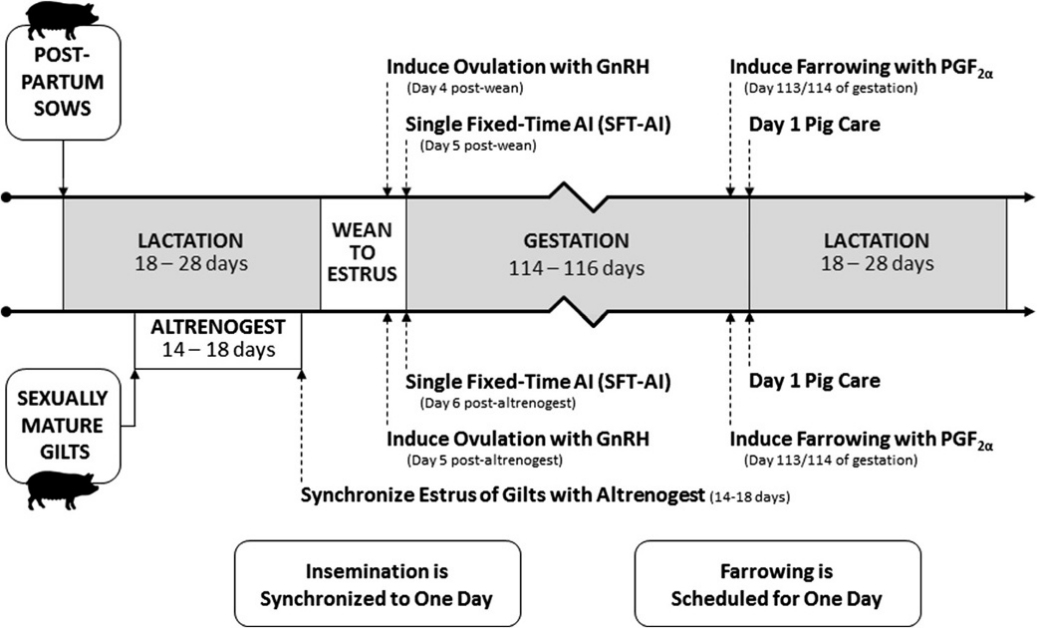
**Model for Synchronizing Breeding and Farrowing in Sows and Gilts.**

Figure 1 presents the authors’ vision for incorporating the discussed technologies into future pig production. The orally active progestin, altrenogest, is used to synchronize the estrous cycle of gilts. GnRH analogs synchronize ovulation thereby making fixed-time AI practical. A single fixed-time AI of every female in a group on one d enables producers to reduce the cost of semen, eliminate weekend inseminations and focus resources on other tasks on the remaining d of the week. Benefits of induced farrowing with PGF_2α_ are: 1) a high proportion of farrowings occur during normal working h, 2) no farrowing on weekends, 3) reduced age and weight range within batches of growing pigs and 4) efficient use of facilities and batching of routine tasks. Ovulation synchronization, single fixed-time AI and induced parturition with PGF_2α_ leads to farrowing synchronization, which facilitates supervision of sows and piglets. Attendance and assistance at farrowing is especially important to ensure adequate colostrum consumption by piglets immediately after birth. These technologies save a significant amount of time, which allows redistribution of labor (i.e. focusing more on facility maintenance, gilt development, evaluating sows’ body condition, adjusting gestation feeders, assisting in farrowing and training workers in the art of stockmanship). In addition, they maximize the leverage of high index boars, which will improve overall pork production efficiency.

The purpose of this paper is to summarize results from basic and applied research that may be applied to management of gilts and sows in the breeding herd and to present the authors’ perspectives on the current strategies actually used by pork producers. Knox and coworkers [5] recently published an analysis of survey data, which documented current reproductive management practices of North American swine farms.

### Gilt development and management

The average sow replacement rate was 45% in 2012 [2]. This high rate is due to failure of postpartum sows to return to estrus and conceive, poor reproductive performance, poor feet and leg soundness, and introduction of new genetic lines [6–9]. Excellent reviews of the literature regarding gilt management were published by Foxcroft [10, 11], Gill [7], Williams et al. [12], Bortolozzo et al. [13], Wiedmann [14] and Whitney and Masker [15]. The authors suggest referring to one or more of these excellent manuscripts for a comprehensive review and discussion on gilt management, development and nutrition since these topics are not discussed in detail in this manuscript.

Breeding and selection of maternal line gilts is generally conducted by breeding stock suppliers based on growth rate, body composition, disease status, sexual development and dam’s reproductive history. The ability to express estrus and continue to cycle should be the key reproductive trait for selection of replacement gilts. Sterning et al. [16] reported that heritability of the ability to display estrus at puberty and ovulate within 10 d after weaning a litter is 0.31. Gilts not displaying estrus at puberty also had a higher incidence of ovulation without estrus within 10 d after weaning their first litter.

Because of the large number of piglets per litter farrowed by modern females, often there are not enough functional nipples for all the piglets. Therefore, the number and functionality of the mammary glands and teats are critical for survival of piglets. Feet and legs are important because sows are expected to farrow more than 2 litters per yr, nurse a large litter for approximately 3 weeks, breed back in 5–7 d after weaning and live on solid concrete or slatted floors [17]. Lameness due to incorrect structure of feet and legs hinder sows from getting up and down in the farrowing crate, which results in reduced feed intake [17, 18].

In general, selected gilts are moved from a growing and finishing facility to a development facility at 150–180 d of age when daily boar exposure begins. Generally age at puberty is positively associated with age at onset of boar exposure [19]. Exposure of peripubertal gilts to boars for 20 min/d stimulates expression of estrus. Boars must be mature (>10 mo of age) and express the full complement of male mating characteristics. For best results, gilts are brought to boars where initially, gilts experience the sight, sound and odor of the boar with fence line contact. Direct physical contact is best. However, constant exposure to boar sounds and scent causes habituation which hinders heat detection, but not necessarily onset of sexual maturity. Gilts expressing estrus are then removed and the boar is allowed full access to all non-cyclic gilts for 10–15 min per d. Moving, mixing, transport and boar exposure typically induce first estrus in a high percentage of gilts within 10–20 d. Gilts that respond to boar exposure at an early age tend to remain in production longer than gilts that respond at a later age [11, 20, 21]. Stimulating gilts to cycle and breed on the second or third estrus is a well-established practice. The terminology, HNS (Heat-No-Serve), is frequently used to describe this important management practice for introducing gilts into the breeding herd.

Cyclic gilts are then moved into the breeding barn for acclimation to facilities and management routines before breeding. Since estrous cycles are known, gilts may be staged into the breeding area to fit into groups of weaned sows. After the first estrus has been recorded, gilts should be acclimated to stalls or breeding and gestation housing at least 16 d prior to breeding.

Most producers breed gilts on the second estrus if they have cycled before 200 d of age, whereas those which express estrus for the first time after 200 d of age are often bred at the first estrus. It is important at first mating that adequate fat stores are available for good lactation and a short WEI represented by a back fat measurement of 12–18 mm. They should be maintained in small groups of approximately 10 with a minimum of 1.4 square meters per gilt [14, 22].

#### Gilt nutrition

Modern maternal line genotypes are more sensitive to nutritional management because their appetite is lower and they have exceptional lean growth potential compared to females of 20 yr ago [10, 23, 13]. Replacement gilts are typically fed ad lib a diet lower in energy than diets fed to slaughter pigs in order to avoid excessive body fat [15]. This also allows for slightly slower growth, which limits mature body size, thereby preventing feet and leg problems and excessive fat gain. An estimate of their genetic potential for growth can be made at this time. Subsequently, diets for replacement gilts should contain higher concentrations of vitamin A and E, calcium, phosphorus, selenium, chromium and zinc than the typical finishing diet because highly prolific gilts reach puberty with limited reserves of protein and body fat and they continue to grow during their first gestation. Concentrations of Ca and P must be high enough for maximum bone mineralization, which is mobilized for fetal growth and lactation [15, 23]. Also, protein and amino acid deficiencies lead to delayed puberty. Older literature indicates that selected replacement gilts should be limit fed energy from 100 to 104 kg BW or until 2 weeks prior to mating so they will not become too fat. However, Foxcroft and coworkers [10], Williams et al. [12] and Gill [7] presented evidence that fatness is not an issue with modern lean maternal line genotype females, which deposit and mobilize lean tissue with little impact on fat tissue depots [10]. Therefore, lean tissue mass is a key consideration for correct management of the gilt [10]. Gill [7] proposed that a nutrition program should result in a body condition score of 3 at first service.

### Sow management

Sow longevity is important because litter size and piglet weights increase until the fourth or fifth parities, and the number of pigs weaned per sow per yr increases until the sixth and seventh parities. Mature, structurally sound replacement gilts will most likely reach their fourth parity, at which time they are most productive for the swine operation [6, 7, 24, 25]. Sow longevity is the number of d from first farrowing to removal from the herd or total number of pigs produced in the lifetime of the sow [21]. Numerous observational studies demonstrated that multiple factors impact sow longevity, such as genetics, nutrition, housing, disease, lameness, age at first mating, assistance at farrowing, length of lactation and growth rate, body condition and performance of parity one sows [20, 21, 26–28]. Specific cultural environments and consumer attitudes in the U.S.A. and Europe influence breeding herd management [14, 29]. Some examples are use of prostaglandin F_2α_ for inducing farrowing_,_ limiting the use of antibiotics and moving away from individual gestation stalls.

#### Sow nutrition

Modern highly prolific females have more defined nutrient requirements than females 20 yr ago [30], thus continuous updating of nutrient requirements and management techniques and technologies are required [10, 23, 30–36]. The effect of environmental factors, such as temperature, humidity and building design on feed intake during lactation should be considered.

*Gestation:* It is well documented that “flushing” by increasing feed by 50–100% or feeding sources of high energy, such as dextrose for 10–14 d before first service, increases ovulation rate and litter size. However, there are conflicting reports in the literature regarding feeding programs during the remainder of gestation [37]. Vignola [38] reviewed research indicating that feed intake should then be decreased after mating to an appropriate gestation diet because sows that are overfed throughout gestation, especially during the first two weeks after breeding, frequently have high embryonic mortality and produce small litters. Sows, which are “too fat”, have farrowing problems, crush piglets, eat poorly during subsequent lactation and are less prolific at the next parity [38]. Sows with back fat depths of 23 mm or more at farrowing have depressed appetite during lactation [38]. Peltoniemi et al. [35] reviewed research indicating that feed restriction after mating may only apply to the first 4 d in gilts and not at all for sows. Love and coworkers [39] and Virolainen and coworkers [40] reported that abundant feeding during early pregnancy increased embryo survival and failed to influence maintenance of pregnancy. Martineau and Badouard [29] proposed that two major characteristics of the hyperprolific sow are lack of early embryonic death with overfeeding after ovulation and a positive influence of overfeeding during the last weeks of pregnancy on piglet birth weights.

As indicated from the above discussion, nutrient needs of sows change significantly as pregnancy progresses. A phase feeding program is used by many producers to accommodate these changes [31, 41–43]. Three stages (i.e. phases of gestation) which justify different feeding strategies are: 1) early gestation (d 0–30), in which embryo survival and implantation are impacted, 2) mid-gestation (d 30–75), in which body growth in young sows and recovery of body reserves lost during lactation in older sows are impacted and 3) late gestation (approximately the last 45 d), in which fetal and mammary growth are impacted. Conceptus protein content and weight gain increases rapidly after d 68 of gestation and has greater priority for nutrient supply than maternal weight gain. Fetal weight, fetal protein content and mammary protein content increase 5, 18, and 27 times, respectively, in the last 45 d of gestation. Therefore, amino acid and energy requirements are greater in late gestation than in early gestation. Amino acid requirements increase to a higher degree than energy requirements in late gestation. If consumption of the same diet increases to meet amino acid requirements, the sows will consume excessive energy, which result in sows being too fat at farrowing. Moehn and Ball [34] recommended a strategy of formulating just two diets; one to meet the highest and the other to meet the lowest amino acid requirements. The two diets would be mixed in appropriate ratios to meet the entire range of amino acid requirements from late gestating gilts to early gestating sows. In practice, feed intake of pregnant sows is actually restricted to control body weight and prevent excess weight gain. Therefore, energy is the limiting factor for gestating sows, and thus, the feed allowance necessary to provide energy requirements must be considered first when formulating a sow feeding regimen. Segregated phase feeding should be considered because maternal growth rate decreases with age so that mature sows have lower nutrient requirements than gilts and young sows, which are still growing.

To regulate feed intake, sows should be fed based on an objective measure of individual body weight, body condition and, ideally, measurement of back fat depth [44]. Feed intake during the last 2 to 3 weeks should be adjusted to at least avoid a negative energy balance prior to farrowing and to promote higher feed intake in early lactation, easier farrowing and adequate birth weights of newborn pigs [38]. However, restriction of feeding prior to parturition significantly reduces the risk of postpartum dysgalactia syndrome (PDS). Peltoniemi and coworkers [35] reported that not only feed restriction before term, but also keeping the feed low in energy and high in fiber through parturition and into the first few d of lactation appears to improve intestinal function and initiation of lactation.

*Lactation:* Highly prolific sows of today produce large litters of lean and fast growing pigs [33, 45]. Litter size increased by three pigs over approximately the last 40 yr [46]. Because appetite is often deficient after farrowing, increased nutrients needed for milk production generally come from mobilization of body reserves [38]. Thus, lactation puts a great nutritional demand on sows. Using body reserves could lead to excessive weight loss, which then results in reduced litter weight gain due to lowered milk production and subsequent reproduction problems for sows. Adequate feed intake, especially during the first 7 to 10 d of lactation is important to replenish body reserves, and re-establish secretion of hormones which control subsequent reproductive performance [47–51]. In addition, numerous studies demonstrated that high ambient temperatures experienced in summer are detrimental to feed intake and milk production [52–57]. Feed restriction at any time results in prolonged weaning WEI and reduced pregnancy rates and litter size. Proper feeder design is critical and has received much attention in recent yr. Most lactation feeders today include a reservoir that will hold a minimum of 9 kg of feed. Ensuring sows have access to full feed 24 h/d results in optimum return to estrus and piglet weight at weaning. Getting sows up 2–3 times per d stimulates sows to urinate and defecate, resulting in drinking and eating, thus optimizing feed intake, lactation performance and return to estrus.

The practice of feeding fat is controversial. This additional source of energy is used principally by the mammary gland to produce very rich milk and it will not be an efficient source of energy for sows [58]. High fat addition could improve piglet weaning weight, but could also impair subsequent reproductive performance by reducing LH secretion in early lactation [51]. Fat as a high density energy source is often incorporated into lactation diets to compensate for depressed appetite during heat stress.

It is essential to have good quality water [59, 60]. Water quality should be checked annually. Lack of water limits milk production. Water available at time of feeding is important with a flow rate of 1.0 liter/minute. High performing sows have a water intake of up to 40 liters/d when milk production is at its highest three weeks after farrowing.

*Post-weaning:* It is well documented that WEI is inversely related to lactation length [61, 62]. Edgerton [61] reviewed research results, which showed that the minimum WEI of 4 d is reached at approximately 3 weeks of lactation. Therefore, each additional d of lactation beyond 3 weeks adds a non-productive d (NPD), resulting in decreased pigs/sow/yr. Mabry and coworkers [63] analyzed records from 178,519 litters in 13 commercial herds around the U.S.A. from 1985–1995. The WEI was minimized at a lactation length of 22–27 d; WEI increased significantly at a lactation length of either less than 22 d or greater than 27 d. Knox et al. [5] reported that, in North America, the most frequently reported average WEI is 5 d and that 88% of farms surveyed indicated that greater than 80% of weaned sows were mated within 7 d. Soede et al. [62] concluded from reviewing the literature that lactation length of less than 3 weeks leads to suboptimal reproductive performance. Currently, sows are weaned at approximately 20.5 to 21 d in the U.S.A. and approximately 22 d in Canada, which is at the height of milk production [3, 64].

Sows experience the stress of piglet removal and change of location, as well as the transition of the mammary tissue into the dry period and follicular development and subsequent ovulation all within 4–5 d. These events require a high level of energy and nutrients. Maintaining ad lib feed and water consumption optimizes these events as measured by subsequent fertility. Boar exposure of sows should start the d of weaning by allowing a boar in front of sows for at least 10 min/d. Weaned sows are typically exposed to boars within 2 d; most commonly once per d [5].

#### Housing and environment

Research on housing and environment was summarized by Einarsson and coworkers [65], Flowers [27], Jansen [66], Kim et al. [33], Rhodes [67], McGlone [68–72], Vignola [38] and Weidmann [14]. Purchased gilts should be quarantined for at least 4 weeks, during which time they should be observed for and serologically tested for infectious diseases. Introduction of young cull sows, market hogs and manure exchange during the latter part of isolation acclimate replacement gilts to the herd’s resident pathogens. Both purchased gilts following quarantine and internally selected gilts should be acclimated in small groups in the breeding barn to allow them to build immunity to organisms present in the breeding herd. Gilts should be vaccinated for diseases, such as E. coli, erysipelas, leptospirosis, mycoplasma and parvovirus, before breeding.

There are conflicting reports in the literature regarding the effects of individual pens versus group housing for gilts and sows. Despite this, consumer attitudes in North America and Europe are persuading regulators to move producers toward group housing of pregnant sows [67, 70–73]. Gilts reared in individual pens or groups of 3 reach puberty significantly later than those reared in groups of 10 or more. In addition, gilts reared in individual pens have more silent heats and irregular estrous cycles than gilts reared in group pens. However, gilts reared in groups of 50 or more also had a lower conception rate than those in smaller groups. In North America, 90% of farms house breeding and gestation sows in standard gestation stalls [5]. In Europe, sows are kept in groups from week 4 of gestation until one week before farrowing [65, 74]. A primary disadvantage of group housing for gestating sows is the inability to uniformly control sow body condition and sow weight gain because dominant sows consume more than timid ones. In addition, aggression of dominant sows causes physical damage to themselves and others. However, feeding stalls with self-locking or manual-locking doors or electronic sow feeders enable sows to have contact with other sows, but have privacy when eating. A stable social hierarchy and well-designed farrowing crates, floors and ergonomic feeders and water nipples, gut fill and lying comfort are important for sow health, performance and longevity. Any stress in the first three weeks of gestation may result in loss of pregnancy or reduced litter size. Moving sows early in gestation should be done gently in small groups.

#### Estrus detection and breeding management

Estrus normally lasts 24 to 48 h in gilts and up to 72 h in sows. Approximately 90% of sows express estrus 3–6 d after weaning [11, 75] and sows which are mated at estrus 4–6 d after weaning have greater farrowing rates and litter sizes than sows mated later than 6 d after weaning [75]. Ovulation occurs approximately 38–48 h after onset of estrus and females ovulate 2/3 of the way through estrus [76–78]. Obviously, because of the variation in time of ovulation relative to onset of and duration of estrus [76, 78–80], the more frequently estrus is checked, the more accurately the time of ovulation can be predicted. Farrowing rates and litter sizes will be lower if insemination occurs more than 24 h before ovulation because sperm live approximately 24 h after insemination and eggs can be fertilized for only 12 h after ovulation [77, 78]. Therefore, the best way to predict ovulation is to detect estrus frequently. Conception rates [78] and litter size [81] are unsatisfactory if mating occurs early or late relative to ovulation. The general practice in the U.S.A. is to inseminate on the d of detected estrus and the morning of the following d.

Failure to detect estrus accurately has the greatest impact on farrowing rate and litter size. Efficiency of estrus detection is significantly lower when gilts are in stalls, or the boar is moved to gilt pens for estrus detection than when gilts are moved to boar pens. Less time is required to elicit standing response and a greater percentage of females are detected when gilts are moved to the boar area than with other methods. Boars must be sexually mature (at least 10 mo of age) and emit odor and sound. Some general recommendations are: check estrus after feeding, remove all distractions from the area, detect estrus in the same place and same way each time, keep animals calm, allow sufficient time for interaction and do not interfere with female and boar interaction.

#### Farrowing management, supervision and induction

Hyperprolific maternal line females of today commonly have 14–16 piglets born alive and piglet pre-weaning mortality ranges from 11 to 24% predominantly in the first five d of age, therefore, there is renewed interest in attendance and assistance at farrowing [45, 82]. Moreover, stillbirth rates increase as litter size increases. In addition, a larger litter generally means smaller and weaker pigs [83, 84]. The rate of stillborn piglets increases as duration of farrowing and interval between births increase. Baxter and Edwards [82] and Vanderhaeghe et al. [85] reviewed literature demonstrating that stillborn pigs are a multifactorial problem, which includes litter size, parity, sow body condition, and farrowing supervision/birth assistance.

Generally, farrowing supervision/birth assistance includes the following practices: 1) preventing savaging of piglets by the sow, 2) manually delivering piglets when the birth interval becomes longer than 30 min, 3) removing placental envelopes around piglets and clearing airways of piglets to prevent suffocation and crushing of piglets, 4) ligating the umbilical cord, 5) towel drying and positioning piglets under a heat lamp immediately after birth to prevent chilling, 6) placing low weight, low-viability piglets in a heated crib or box away from the sow, 7) feeding low-viability pigs colostrum or milk replacer orally if necessary, 8) “split suckling” or cross-fostering litters to ensure piglets from large litters consume adequate colostrum, 9) administering fluids to dehydrated piglets, either orally or subcutaneously, and 10) taping legs of splay-legged piglets together. Many producers practice the McREBEL^™^ management program, which minimizes cross-fostering and maximizes supportive care [86–88]. The most important factor for ensuring piglet survival is to ensure adequate colostrum consumption immediately after birth, particularly since colostrum production by the sow occurs for only 24 h after farrowing [89].

Several controlled studies have investigated the benefits of farrowing supervision/birth assistance. Holyoake et al. [90] assigned sows and gilts to 4 treatments in a 2 × 2 factorial arrangement: 1) induced/supervised, 2) non-induced/supervised, 3) induced/unsupervised and 4) non-induced/unsupervised. Farrowing was induced with 250 μg of PGF_2α_. Each supervised group was supervised from 3 h before the first expected farrowing time until the youngest litter of pigs in the group was 3 d old. Therefore, litters born first within a group were supervised for longer than 3 d. Number of stillbirths/litter and number of pre-weaning deaths/litter were significantly greater (*P* < 0.05) for unsupervised sows (0.68 ± 0.08 and 1.29 ± 0.13, respectively) than for supervised sows (0.26 ± 0.08 and 0.86 ± 0.13, respectively), whereas total weaned/litter was less (*P* < 0.05) for unsupervised sows (9.44 ± 0.19) than for supervised sows (10.17 ± 0.2). Of the 274 piglets which died in the pre-weaning period, 47% died during the first 3 d after birth and 62% died during the first 4 d after birth. White et al. treated 30 sows each as follows: Group 1 (Control)–farrowings were not attended and Group 2–farrowings were attended and piglets assisted on the d of and the d after farrowing. The percentage of stillborn piglets was 6.8 for Control sows and 1.6 for Group 2 sows (*P* < 0.05). Overall, preweaning mortality was 18.2 for Control sows and 10.1 for Group 2 sows (*P* < 0.05). Nguyen et al. [91] assigned multiparous sows to 2 treatments: Group 1 - farrowing was induced with PGF_2α_ and farrowing was supervised and piglets were assisted as needed on the d of farrowing only and Group 2 - farrowing was not induced and not supervised. The percentage of stillbirths was lower (*P* < 0.001) for Group 1 (0.4 ± 0.09/litter) than for Group 2 (1.0 ± 0.17/litter). Twenty-seven percent of Group 1 sows had at least one stillborn piglet whereas 49% of Group 2 sows had at least one stillborn piglet. However, there was no effect of treatment on overall pre-weaning mortality. Nguyen and coworkers [91] concluded that sows and piglets require more than one d of supervision to reduce overall pre-weaning mortality.

#### Seasonal infertility

The effect of season on fertility is mediated by temperature and photoperiod [92–97]. Puberty is delayed in summer mo and the WEI and duration of estrus are longer and ovulation rate, conception rate and litter size are lower in summer than in late autumn and winter. Parity one sows are more susceptible to reduced reproductive performance than older sows.

As noted by Claus and Weiler [93], photoperiod is the only environmental factor which is highly repeatable from yr to yr. Artificially altering photoperiod failed to influence WEI, conception rate, farrowing rate or litter size when light/dark ratios were abruptly changed and held constant [97, 98]. Kraeling and coworkers [99] reported that exposure of lactating sows and ovariectomized gilts to 8 h light/16 h dark or 16 h light/8 h dark failed to affect prolactin, growth hormone or luteinizing hormone secretion. Nevertheless, when photoperiod was extended to 16 h light/8 h dark, milk yield increased by 20–24%, thus piglet survival rate and body weight improved, which was explained by differences in suckling frequency of the piglets [100]. Pigs may not be able to respond to sudden changes in photoperiod. However, Auvigne and coworkers [101] analyzed ultrasound diagnosis results from farms located in four regions of France for 5 yr (2003–2007). Seasonal infertility was significantly higher during 2003 than in the other four yr, which did not differ among each other. In all regions, the highest number of hot d was in 2003 with the least number of hot d in 2007. They concluded that photoperiod has a prominent role in seasonal infertility with an additional influence of heat stress during the hottest yr.

High environmental temperature decreases lactation feed intake, delays puberty, disrupts behavioral estrus, lowers ovulation rate, increases embryonic mortality, decreases milk production and prolongs the WEI in sows. Heat stress is most detrimental to reproductive performance during the first 30 d due to increased embryonic death and last 30 d of gestation due to increased stillborn piglets. Management and nutrition determine the degree of impact of season on reproduction. Strategies to reduce heat stress are: 1) feed high energy diets with lower fiber and crude protein content, 2) feed at night, 3) feed multiple times per d, 4) use air cooling or water dripping equipment, and 5) decrease group size to 15 or fewer in gestation and use individual gestation stalls to reduce social stress. Most farms in North America experience seasonal infertility caused by estrus failure in gilts and weaned sows and pregnancy failure [5].

Photoperiod may modulate the impact of other management factors unless it is extremely skewed to either all light or all dark, but by itself photoperiod likely has a minimal impact and is not a major factor in seasonal infertility. Decreasing photoperiod and high temperatures generally occur at the same seasonal time frame. The wild boar is not selected for continued reproduction, yet remains a seasonal breeder [93, 96]. To optimize sow production producers should manage sow herds to minimize heat stress and adapt light and dark cycles to avoid either excessive light or dark periods.

### Stockmanship

Hemsworth and coworkers [102, 103] reported that pigs, which displayed a high level of fear of humans, had sustained elevation in plasma concentrations of corticosteroids associated with poor conception rate and litter size. In a study of 19 commercial farms in Australia, there were highly significant negative correlations between sows’ level of fear of humans and reproductive performance of the farm, and the stockperson’s behavior was significantly correlated with both the sows’ level of fear of humans and productivity of the farm. Kirkden and coworkers [104] examined the effect of the stockperson’s skill and attitude on reproductive performance. As expected, most studies showed that positively handled pigs are less fearful of humans than pigs exposed to electrical prod and that occasional negative experiences have a significant impact on the way pigs perceive the stockperson. Sows which are fearful of humans during gestation are more likely to savage their piglets and repeated aversive handling of sows during late gestation results in increased piglet morbidity. Training farm workers in the art of stockmanship continues to be a challenge for swine farm managers and is of increasing importance as consumers become more interested in humane farm animal care.

### Strategies for use of new and current management technologies

Knox [75] and Estill [105] reviewed the impact of reproductive technologies on pig production. These technologies dramatically changed the way pigs are raised and made the pig the most efficient livestock species for food production in the world. Many of the technologies developed during the past 2–4 decades have been incorporated into modern pork production systems. In many cases, producers have adapted and are utilizing the technologies for applications or objectives that differ from the original or approved use or claim. The authors’ intent is to describe and discuss how the technologies are actually being applied and not to endorse or advocate product use that may differ from the regulatory approval in various countries.

#### Altrenogest

*Gilts:* The search for an effective and acceptable method to synchronize estrus and ovulation in post-pubertal gilts began over 50 yr ago [106]. The corpus luteum (CL) of the pig has an inherent life span of 14–16 d [107, 108] and is resistant to the luteolytic action of uterine PGF_2α_ secretion before d 12 of the estrous cycle [109, 110], thereby making PGF_2α_ ineffective for estrous synchronization. Therefore, the predominant approach to estrous synchronization in the gilt was to administer a treatment, which suppresses pituitary gonadotropin secretion for 14 to 20 d to allow time for the CL to regress, and at the same time, prohibit growth of new follicles and ovulation. Upon withdrawal of such a treatment, it was expected that gonadotropin secretion would resume synchronously among the treated animals. Orally active, synthetic progesterone-like compounds were the most commonly investigated [111]. Unfortunately, the post-treatment estrus was often accompanied by development of ovarian cysts, decreased fertility and/or poor synchronization of estrus and ovulation. However, based on exhaustive studies of the progestin, altrenogest (17α-allyl-estratiene-4-9-11,17-β-ol-3-one), in the 1970s and 1980s, as reviewed by Webel and Day [111, 112] and Estill [105], the Food and Drug Administration approved its use for estrous synchronization in sexually mature gilts in 2003 (Federal Register, October 31, 2003). Altrenogest is marketed by Merck Animal Health under the trade name, MATRIX® in the U.S.A. and by several other companies under other trade names outside of the U.S.A. In controlled studies [111, 113–115], and in applied studies at large commercial farms in the U.S.A. [116, 117], approximately 85% of gilts fed 15 mg of altrenogest/gilt/d for 14 d displayed estrus within 4 to 9 d after withdrawal of altrenogest. For maximum effectiveness, post-pubertal gilts must have displayed at least one estrus before feeding altrenogest.

*Sows:* Because farrowing time within a group of sows is spread over as much as a 10 d period, “batch farrowing” and “all-in-all-out” production practices are facilitated by delaying parturition in the sows which were mated earliest in the group and inducing farrowing in those mated later in the group. Allowing pigs to stay in utero an extra 2–3 d improves birth weight and colostrum antibodies increase in the sow as gestation length increases. Farrowing on weekends is avoided when parturition is precisely controlled. Guthrie [118] noted that the effectiveness of an orally active progestogen to delay parturition in the sow was reported over 50 yr ago. Numerous researchers demonstrated that intramuscular injections of progesterone or feeding altrenogest for 2–3 d, starting several d before the time of normal parturition, prolongs gestation without affecting the incidence of stillbirths, piglet mortality or dystocia in the sow [118–122]. However, to prevent increased stillbirth rates, the length of gestation should not be prolonged by more than two d beyond the normal herd average. Guthrie et al. [121] demonstrated that farrowing is even more precisely synchronized after administration of PGF_2α_ the d after the last progestogen treatment.

Numerous studies demonstrated that postponing post-weaning estrus by administering altrenogest improves reproductive performance [73, 123–125]. Although not approved for this use in the U.S.A., feeding altrenogest for 5–7 d delays onset of postpartum estrus, which gives extra time for sows to recover body condition lost during lactation, to establish batch farrowing groups and to establish sow groups following piglet death from diseases such as porcine epidemic diarrhea virus (PEDV). In addition, subsequent conception rate and litter size increase. The most prevalent use of altrenogest in commercial herds is for delaying early farrowing before d 115 of gestation and for the transition from continuous to batch farrowing. Typically, producers employ altrenogest for delaying estrus in weaned sows for one to two weeks to assemble groups or batches of sows.

#### Gonadotropins

*Prepubertal and peripubertal gilts:* The only commercially available hormone preparation for inducing estrus and ovulation in prepubertal gilts and postpartum sows in the U.S.A. is P.G. 600®, each dose of which contains 400 IU of pregnant mare’s serum gonadotropin (eCG) and 200 IU of human chorionic gonadotropin (hCG). Numerous studies demonstrated that i.m. injection of P.G. 600® in prepubertal and peripubertal gilts induces estrus in 50–90% within 5 d [73, 77, 126–130]. Up to 30% of those exhibiting estrus have an irregular return to a subsequent estrus.

*Postpartum sows:* Many studies demonstrated that i.m. injection of PMSG or P.G. 600® in sows at weaning induces estrus in sows within 5 d [73, 77, 126, 128, 130–132]. Weaning to estrus interval is shorter, but the estrus synchronization rate, subsequent farrowing rate and litter size are similar compared to untreated sows. Gonadotropins are often misused on production farms by attempting to induce estrus in sows or gilts in the presence of CL or perhaps cystic follicles. For example, treating sows that have not expressed estrus by 10–12 d following weaning is a common practice, but is often ineffective because many sows experienced a silent estrus and the gonadotropin treatment is ineffective. The most common and effective use of gonadotropin treatment is for parity one sows and during seasonal anestrous. For inducing estrus, P.G. 600® is typically given on the d of weaning to induce a more synchronous return to post-weaning estrus.

#### Fixed-time artificial insemination (AI)

As noted above, feeding 15 mg of altrenogest/gilt/d for 14 d results in approximately 85% of the gilts displaying estrus within 4–9 d after withdrawal of altrenogest. Weaning a group of sows, when all have reached three weeks or greater of lactation, results in a high percentage of these sows being bred within a 3 d period beginning about 4 d after weaning. Therefore, a treatment, which more precisely synchronizes ovulation, is needed to facilitate a single fixed-time AI of all gilts and sows in a group on the same d without regard to onset of estrus, thereby eliminating the need for estrus detection.

Brüssow et al. [128] and Driancourt [133] summarized literature which demonstrated the effectiveness of hCG, LH and GnRH analogs to synchronize ovulation in weaned sows and in gilts after withdrawal of altrenogest. Zak et al [134, 135] reported that i.m. administration of 5 mg of pLH to weaned sows at onset of behavioral estrus followed by a double fixed-time AI resulted in a farrowing rate comparable to controls inseminated multiple times while in estrus. JBS United Animal Health launched OvuGel®, a FDA licensed proprietary gel formulation containing a GnRH-analogue in 2013. OvuGel® is the first product approved for synchronizing ovulation followed by a single fixed-time AI in weaned sows. OvuGel® is administered intravaginally to sows 96 h after weaning. Because ovulation starts in some sows 32–36 h after OvuGel® administration and a high percentage of sows complete ovulation 40–48 h after OvuGel® administration, all sows are inseminated with a single dose of semen without regard to estrus 22–24 h after OvuGel® administration to optimize fertility [136]. Thus, there is no need for heat detection, which effectively decreases labor costs and increases throughput and utilization of inventory. Fertility after OvuGel® treatment followed by a single fixed-time AI are equivalent to those of untreated sows inseminated on each d in standing estrus [137].

Driancourt and coworkers [133, 138] administered buserelin acetate 115–120 h after last feeding of altrenogest in gilts and 83–89 h after weaning in sows via intra-muscular or subcutaneous injection. A single AI was performed 30–33 h after buserelin treatment in only females which had displayed estrus. Fertility was equivalent to those of untreated animals.

Time of farrowing among a group of sows, all of which receive a single fixed-time AI on the same d is less variable than those receiving a AI on each d they are in estrus. This breeding precision facilitates a synchronized farrowing for the majority of a breeding group, which provides an opportunity for careful attention to d one pig care and reduction in stillborn rates. An even more precise synchronized farrowing is achieved by induction of parturition with prostaglandin F_2α_.

#### Cervical artificial insemination (CAI) and post-cervical artificial insemination (PCAI)

Cervical artificial insemination (CAI) is the predominant breeding method on farms of all sizes [5]. Benefits of CAI and PCAI are introduction of improved genetics, reduced risk of disease transmission and improved performance of reproductive tasks, which improves time management compared to natural service [139]. CAI technician fatigue should be avoided. Farrowing rates decrease from 85 to 78% when technicians perform more than 10 CAIs before taking a break. Farrowing rate decreases to 71% when more than 15 CAIs are performed without a break.

Recent interest in PCAI is primarily because sperm cell numbers/dose of semen is significantly less than that of CAI [140–142]. Therefore, more sows are inseminated with superior genetics and fewer sires are required to produce such semen. PCAI bypasses the cervix and deposits the majority of semen directly into the uterine body. PCAI, also known as intrauterine AI and trans-cervical AI, is not new [143–145]. Some reported disadvantages of PCAI are that it is not easily applied to gilts, the catheter is expensive and it could be harmful to the female, if not performed correctly. The benefits are reduced labor, decreased time performing AI, more sows bred with semen from superior sires and fewer sires needed to produce that semen [140–142]. Additional time and labor are saved because no boar should be present during PCAI. Traditional CAI takes 7–10 min, whereas PCAI takes one to two min. With PCAI, 1–2 × 10^9^ sperm cells/dose [140, 142] or even 0.5 × 10^9^ sperm cells/dose [146] are used compared with 3 × 10^9^ sperm cells/dose used for CAI.

Several workers reported that reproductive performance was compromised after PCAI in primaparous sows [147]. However, Sbardella and coworkers [148] compared the reproductive performance of primaparous sows, which were mated by PCAI with 1.5 × 10^9^ sperm cells in 45 mL, and those which were mated by cervical AI with 3 × 10^9^ sperm cells in 90 mL. There was no difference between treatments in farrowing rate and litter size. Passage of the intrauterine catheter was possible in 86.8% of the PCAI sows. PCAI is particularly beneficial when using frozen-thawed semen and sex sorted sperm, which reduce the number of sperm cells available and/or the lifespan of sperm cells.

Knox et al. [5] documented the following practices for farms in North America. Fifty-five percent of farms used the interval from weaning to estrus to time the first AI, after detection of estrus, 62% of farms timed the first AI to occur within min or a few h of estrus, whereas 30% delayed AI until the next AM or PM period. Seventy-six percent of farms planned for two doses of semen for each sow, whereas only 14% planned for three doses of semen per sow. Prominent procedures during AI were back pressure (93%), boar exposure (89%), flank rubbing (80%) and gravity semen flow (81%). PCAI is practiced on only 6% of farms; 61% having no experience with PCAI and 25% having tried it, but not used it since. However, during the past two yr, interest in and implementation of PCAI has experienced a dramatic increase with a high success rate and may soon become the most prevalent AI technique.

#### Ultrasound for pregnancy detection

According to Flowers and coworkers [149] and Knox and coworkers [73], the most common strategy for identifying non-pregnant females is detection of estrus by daily boar exposure from 17 to 23 d after breeding followed by examination by either amplitude-depth (A-mode) or Doppler ultrasonography between d 28 and 45 of gestation. In addition, Flowers and coworkers presented data demonstrating that real-time (B-mode) ultrasonography is accurate when used after the first 3 weeks of gestation. In North America, most medium and large farms use real-time ultrasonography to determine pregnancy during week 3 to 5 [5].

#### Induced farrowing

Guthrie [118], Kirkden and coworkers [104, 150], Kirkwood [129] and Nguyen et al. [91] published literature reviews on the impact of induction of synchronized farrowing on piglet mortality. Farrowing is usually induced by administration of PGF_2α_ or a PGF_2α_ analog. Numerous studies indicate approximately 92% of sows farrow within the working d following PGF_2α_ injection compared to 38% for untreated controls. Induction of farrowing substantially decreases piglet mortality because 1) a high proportion of farrowings occur during normal working h, 2) farrowing can be closely supervised, which provides opportunity to save and cross-foster piglets, 3) farrowing is avoided on weekends, 4) batch farrowing reduces variation in piglet age at weaning, and 5) batching of routine tasks result in efficient use of facilities. Weaning a group of piglets at a more narrow age range, results in less variation in subsequent market weights. The negative experiences some producers have had with induction of farrowing were most likely due to incorrect use of PGF products. PGF_2α_ must be administered no earlier than 2 d before expected farrowing based on the average expected farrowing date of the herd, since mean gestation length between herds varies from 113 to 117 d. Induction too early will result in low piglet birth weight, increased duration of farrowing and increased stillborn and live born mortality rates. Induction of farrowing has not been widely practiced in the USA, except for inducing sows with the longest gestation to ensure they farrow with a particular group.

Generally, natural farrowing time within a group of sows is spread over approximately a 10 d period due to variation between sows in weaning to estrus interval and length of gestation. However, due to increased interest in biosecurity and piglet health issues, U.S.A. producers have expressed renewed interest in “batch farrowing” and “all-in-all-out” production practices. The introduction of ovulation synchronization and single fixed-time AI inspired a vision for closer farrowing synchronization to facilitate supervision and reduce stillbirths. If a “batch” or group of sows are induced to ovulate and inseminated once at the same h on the same d, then farrowing induction with reduced time and labor required for providing supervision for the batch becomes practical. Recent trials support this theory in that the time and variability of farrowing was reduced for sows inseminated following treatment with OvuGel® and a single fixed-time AI compared to contemporary control sows inseminated without ovulation synchronization [149]. Furthermore, addition of PGF_2α_ to induce farrowing on d 113 of gestation resulted in highly synchronized farrowing, reduced variation in and increased age of piglets at weaning. Greater than 80% of the sows inseminated with a single fixed-time AI, then treated with PGF_2α_ on the same d (113 of gestation), farrowed on the same d. Ninety percent of the piglets were the same d of age at weaning and were 1.3 d older than those from non-induced sows. In these studies, 92% of treated sows farrowed within 2 d compared to 38% for controls [151, 152]. The precision observed following single fixed-time AI and treatment for induction of farrowing on the same d now permits farrowing managers to attend farrowing and provide intensive piglet care. As the practice of batch farrowing, fixed-time AI and intensive birthing care become more prevalent, the pre-weaning survival of piglets is expected to increase. Figure 1 schematically depicts the authors’ vision for utilization of currently available technologies. These combined technologies will result in production benefits discussed above.

### New performance terminology

Figure 1 schematically depicts the authors’ vision for utilization of currently available technologies. These combined technologies will result in production benefits discussed above. Because all weaned sows are inseminated once (Figure 1) without regard to estrus in a fixed-timed AI program, the conventional farrowing rate terminology of number farrowed ÷ number bred is not appropriate. Webel and coworkers [151] suggested that weaned sow farrowing rate (number farrowed ÷ number weaned) is a more appropriate measure of sow utilization when comparing farrowing performance. Also, the commonly used metric of pigs per mated female may be a misleading indicator of sow farm productivity because it does not account for sows that do not return to estrus and are not mated promptly following weaning. For a group of weaned sows, total live pigs produced per 100 weaned sows (piglet index) provides a more valuable economic measure of sow farm efficiency [151, 152].

## Conclusion

Many advances in genetic selection, nutrition, housing and disease control since the 1950s have been incorporated into modern pork production. Genetics, nutrition, housing, disease, lameness, age at first mating, assistance at farrowing, length of lactation and growth rate, body condition and performance of parity one impact sow longevity. Seasonal infertility, mediated by temperature and photoperiod, is a persistent problem. The following technologies have been adopted by many swine producers over the past 2–4 decades. The orally active progestin, altrenogest is used to: 1) synchronize the estrous cycle of gilts, 2) prolong gestation to synchronize farrowing and 3) postpone post-weaning estrus to give extra time for sows to recover body condition lost during lactation. The only commercially available preparation for inducing estrus and ovulation in the U.S.A. is P.G. 600®_._ GnRH analogs synchronize ovulation thereby making fixed-time AI practical. A single fixed-time AI of every female in a group on one d enables producers to plan precisely how much semen to have available on a particular d and to focus resources on other tasks the other 6 d of the week. It is also possible to eliminate weekend inseminations, and if breeding is performed on a weekend, the AI technician knows exactly what needs to be done, thereby reducing errors. Therefore, less semen is wasted and less old semen is used from previous orders. Post-cervical AI uses significantly fewer sperm cells/dose of semen than cervical AI. Real-time ultrasonography is used to determine pregnancy during weeks 3–5. Benefits of induced farrowing with PGF_2α_ are: 1) a high proportion of farrowing occur during normal working h, 2) close supervision at farrowing, 3) no farrowing on weekends, 4) reduce age range within batches of growing pigs and 5) efficient use of facilities and batching of routine tasks. Ovulation synchronization, single fixed-time AI and induced parturition with PGF_2α_ leads to farrowing synchronization, which facilitates supervision of sows and piglets. Attendance and assistance at farrowing is especially important to ensure adequate colostrum consumption by piglets immediately after birth. These technologies save a significant amount of time, which allows redistribution of labor (i.e. focusing more on facility maintenance, gilt development, evaluating sows’ body condition, adjusting gestation feeders, assisting in farrowing and training workers in the art of stockmanship, which is important for humane farm animal care). In addition, they maximize the leverage of high index boars, which will improve overall pork production efficiency. New performance terminologies were proposed. Because all weaned sows are inseminated once without regard to estrus in a fixed-timed AI program, the conventional farrowing rate terminology (number farrowed ÷ number bred) is inappropriate. Weaned sow farrowing rate (number farrowed ÷ number weaned) is a more appropriate measure of sow utilization when comparing farrowing performance. Also, the commonly used metric of pigs per mated female is a misleading indicator of sow farm productivity because it does not account for sows that do not return to estrus and are not mated promptly following weaning. Therefore, total live pigs produced per 100 weaned sows (piglet index) provides a more valuable economic measure of sow farm efficiency.
